# Factors that determine the level of participation in sport and exercise—an analyze of public policy for sport and exercise in European countries

**DOI:** 10.3389/fspor.2025.1633869

**Published:** 2025-10-24

**Authors:** Nils Asle Bergsgard

**Affiliations:** Department of Sports, Physical Education and Outdoor Studies, University of South-Eastern Norway (USN), Bø, Norway

**Keywords:** policy, sport and exercise, participation, government, contextual factors

## Abstract

**Introduction:**

The burgeoning issue of physical inactivity on a global scale, prominently underscored by the World Health Organization (WHO) and the European Union (EU), poses significant health challenges. This necessitates a concerted effort to elevate levels of physical activity. This paper delves into the public policies concerning sport and exercise within EU countries to identify pivotal factors that encourage sports and exercise participation.

**Methods:**

Employing data from Eurostat, Eurobarometer, and Eurofound, this study investigates the patterns of sports participation across Europe. The analysis encompasses descriptive statistics, correlation analyses, and multivariate regression models, with a focus on variables such as economic prosperity, educational attainment, and governance quality.

**Results:**

The findings reveal a weak correlation between public expenditure on sports and participation rates. Interestingly, Eastern and Southern European countries allocate a proportionally higher share of their budgets to sports yet report lower participation rates. Moreover, the quality of governance and overall economic conditions emerge as important factors, displaying a robust association with participation rates and eclipsing the potential impact of direct government spending.

**Discussion:**

The study highlights the limited direct impact of public sports policies, emphasizing the pivotal role of contextual factors in shaping participation rates. Socioeconomic elements such as wealth, education, and governance emerge as critical drivers. Cultural dynamics, often underestimated, prove to be a vital factor in fostering engagement. Furthermore, effective governance and comprehensive welfare systems appear to indirectly bolster participation, creating an environment conducive to more active lifestyles.

**Conclusion:**

To enhance exercise and sports participation across Europe, it is essential to adopt integrated policies that go beyond sport-specific initiatives, addressing the broader cultural, economic, and infrastructural dimensions.

## Introduction

Studying leisure time activities, particularly sports and physical activities, is important due to their perceived benefits that span both intrinsic and extrinsic dimensions. Intrinsically, these activities can foster enjoyment, enhance well-being, and create a sense of belonging. Extrinsically, they can facilitate integration within communities and significantly contribute to mental and physical health improvements. The significance of these extrinsic benefits, especially the health aspect, has been underscored by major organizations like the World Health Organization (1) and the European Union (2). The issue of physical inactivity among populations is thus a pressing concern, given its association with the rise in obesity and other non-communicable diseases. A pivotal study conducted by Strains et al. (3) reveals that approximately one-third of the global adult population (aged 18 and above) had insufficient physical activity levels in 2022. Alarmingly, this figure has increased by 8 percentage points since 2000.

Focusing on Europe, the study (3) indicates a decrease in inactivity rates in high-income western countries, contrasted by a rise in central and eastern European regions. Despite these trends, neither region inside Europe is poised to meet the global target of a 15% reduction in physical inactivity by 2030. A recent analysis by Ritchie et al. (4) examined the social framing of physical activity within EU Member States' policy documents. It identified health, predominantly physical and mental health, as the primary theme. Although other themes like social and community engagement, environmental considerations, and overall well-being are present, they are less emphasized. The authors advocate for a broad social framing of physical activity to engage a diverse array of stakeholders in its promotion, thereby enhancing its uptake and impact [(4), p. 7].

The promotion of sports and physical activities is widely regarded as essential, not only for enhancing individual health but also for strengthening community cohesion and advancing broader societal objectives. Against this backdrop, this paper opens by posing the central research question: What role do public policies for sport and exercise play in shaping participation levels across European countries? It further explores a secondary question: What is the potential impact of other variables in accounting for the observed disparities in participation rates between nations?

What do we mean by sport and physical activity? For the purposes of this study, we adopt the definition provided by the European Council in 1992, which encompasses both recreational and competitive sports but excludes physical activities linked to work or study, such as walking or cycling to work or school. This paper focuses on examining the role of the public sector in promoting sport and exercise, as well as other potentially relevant factors, in explaining the observed variations in participation levels across countries. Consequently, throughout this paper, the term *sport and exercise* will be used, aligning with the terminology of the dependent variable analyzed in this study (as detailed below). However, on occasion, alternative phrases such as “sport activities” or “sport policy” may be employed; in all cases, these terms refer to *sport and exercise* as defined above.

### Background

#### Variation in region and welfare systems

Esping-Andersen's (5) tripartite classification of Western welfare regimes, initially introduced in 1990, has since undergone significant refinement and elaboration [for further details see, for example (6), pp. 6–8 (7–9)]. According to this, it can be reasonably asserted that there is a degree of overlap between the characteristics of welfare regimes and the geographical regions in which they are situated. The conservative welfare regime, which is characterized by the linkage of welfare benefits to occupation and corporatist status division, and in which the non-profit/voluntary sector provides a significant proportion of welfare services, is a prominent feature of many Western European continental countries. The social democratic welfare regime is typified by a dominant state role in providing welfare services and social guarantees, as well as universal and generous social benefits. This is characteristic of Northern European countries. The classification of nations as either conservative (advanced Christian democratic) or social democratic welfare regimes is not a simple matter. Arcanjo (9) employed Esping-Andersen's methodology to analyze the political orientations of several European nations, and identified the Netherlands, Belgium, and Austria as social democratic countries, while Finland was classified as conservative. Furthermore, Ireland is the sole country in the statistics that can be categorized as Anglosphere countries with liberal welfare regimes. The Latin Rim, comprising the nations of Southern Europe, can be characterized as a hybrid model blending elements of both liberal and conservative welfare regimes, with a pronounced emphasis on the family as the cornerstone of social welfare provision.

The designation of the former Eastern Bloc countries, which are grouped here as Central and Eastern European countries, is not clear. In previous research, these countries have been described as 'state-bureaucratic welfare regimes', a term that refers to the former communist ideology [(6), p. 7]. However, these countries have followed disparate paths over the past three decades. Consequently, it is not pertinent to identify their welfare regimes with a single label.

#### Welfare regimes and governments sport policies

It is also interesting to see how government involvement in sport can vary, and often not in an expected way. In the social democratic welfare regimes, e.g., the Nordic countries with large public sectors, government funding is in the upper half, as identified in the figures below. There is still less governing of the sports sector, which is by and large driven by voluntary organizations or private fitness centers. While in liberal welfare regimes such as the UK, Canada and New Zealand, the focus is on management by objectives, especially for elite sport, but also for national sport governing bodies (6, 10).

Nicholson et al. conclude, in a volume with contributions from 16 countries within and outside Europe, “sport participation rates do not appear to be correlated to a nation's sport structure or delivery system” [(10), p. 295]. They further comment: “It is evident from the vast majority of the chapters within this book that government policies designed to increase sport participation have limited success” (p. 305). This is also supported by a study of the relationship between government and federations in 13 European countries: “In conclusion, huge cultural differences and differences in policy systems, even within the Western world, make any international comparison somewhat risky because any sport system is both culturally and contextually bound” [ (11), p. 318].

#### The structure of the sports sector

The structure and composition of the sports sector exhibit considerable variation across countries. For example, nations like Greece, Italy, and Portugal predominantly foster participation through sports centers, whereas countries such as Denmark, the Netherlands, and Austria rely heavily on club membership to drive engagement (12). Despite these national differences, a unifying trend across Europe is the predominance of informal sports participation, with the majority of activities occurring outside formal organizational frameworks in the form of self-organized sport and exercise. A comparative study analyzing the characteristics of sports clubs in ten European countries (13) found no consistent patterns tied to regional or political systems. However, several notable trends emerged: sports clubs in Northern and Western Europe, including Germany and Switzerland, tend to be larger in size than those in Central and Southern Europe. Furthermore, clubs in Central and Northern Europe receive the highest proportion of their income from public subsidies, while those in Central and Southern Europe, such as Spain, are more dependent on access to public facilities rather than owning their own infrastructure.^1^

While conducting comparative studies of sport policies across countries carries inherent risks, as highlighted earlier, we will endeavor to address both country-specific and cross-country factors. This approach aims to identify key elements that contribute to explaining variations in participation rates in sport and exercise.

## Method—materials, measures and analyses

### Materials

The analyses presented in this paper are based on data from Eurostat, Eurobarometer, and Eurofound. The data was gathered as part of the Public Sector Performance Programme (2022–2025), an International Benchmark Study, Sub-Study 2024, conducted by the European Institute of Public Administration. These data sources draw on public records, such as funding for sports and recreation, as well as statistical surveys conducted within EU member states (*N* = 27). However, it is important to interpret these statistics with caution, as their quality can vary significantly. In some cases, particularly for certain countries, the reliability of the data may be low. This concern applies not only to public records but, more critically, to surveys conducted at the national level. For instance, issues of both reliability and validity may arise due to differences in how respondents interpret terms like “play sport”—a point we will elaborate on later. That said, our analysis focuses not on individual countries but on the broader patterns that emerge across them. As such, while issues of reliability or validity may affect specific cases, the overall trends and the key factors influencing sports and exercise participation levels should remain relatively consistent across the dataset.

### Measures

We employ three distinct metrics to examine public expenditure on sport and recreational services. First, we consider the proportion of general government expenditure as a percentage of GDP. This provides a broad indicator of how sport and recreational services are prioritized relative to the overall economy. Second, we look at the expenditure as a percentage of total government spending. This measure offers a different perspective because it is relative to the overall government expenditure. For example, a country might prioritize sports highly compared to other sectors, but if its total public spending is relatively low, as in some liberal economies, the percentage of GDP allocated to sports will also be low. The third metric is the expenditure in Euro per inhabitant, which partly accounts for the general wealth of each nation.

It is essential to understand the scope of the category “sport and recreational expenditure” as defined by Eurostat. Recreational and sporting services include (COFOG Group 08.1)^2^ expenditure on the administration of sports and recreational affairs, on sports facilities (such as playing fields, tennis courts, running tracks, gymnasia, etc.)—including support, operation, supervision, and regulation of those facilities—on facilities for recreational purposes (such as parks and swimming pools), and on grants, loans, or subsidies to support teams, individual competitors, or players.

For the outcome variable, we have selected a specific metric—exercise or playing sport—as it effectively encapsulates the patterns observed across various other metrics. This choice is further elaborated upon in the following section. The relevant Eurobarometer question asks: “How often do you exercise or play sport? By “exercise,” we mean any form of physical activity that you do in a sport context or sport-related setting, such as swimming, training in a fitness center or sport club, or running in the park.” Naturally, there are some challenges with self-reported answers, as respondents often tend to slightly exaggerate their activity levels (14). It is difficult to determine whether this potential bias follows any structured patterns across countries. However, as argued in the next section, there may be cultural differences in how concepts like “play sport” are interpreted, which could influence responses.

### Difficulties measuring between various countries

In an update of the COMPASS project, which compared participation in sport for seven European countries, the authors wrote: “The difficulties of comparing sports participation data collected in different countries have long been recognized, as have the potential benefits to sports administrators and decision makers of having access to comparable statistics” [(15), p. 99; see also (16)]. As Van Tuyckom et al. (12) emphasize, the reliability of studies is not the only issue that must be considered. The validity of studies is also of great importance, as it concerns the interpretation of concepts such as “sport” and “physical activity” and the understanding of related terms in different countries. The issue of reliability is partially addressed here since the statistics employed encompass all countries. However, the manner in which this is conducted in each country may still vary [see (17)].^3^ Further, the challenge of validity—the lack of equivalence of meaning—is still relevant [see (18)]. For example, Stamm and Lapmrecht (19) relate the discrepancies in the participation rate between the linguistic regions of Switzerland to the varying definitions of sport within the national and cultural context. Moreover, the authors posit that the expansion of the concept of sport in Western European countries over the past 20–30 years, while a more “conservative” interpretation in Eastern and Southern European countries, may account for some of the observed discrepancies in participation rates.

It is therefore evident that contextual factors, such as culture, civic traditions, history, and climate play a significant role in explaining the observed variations in sports and physical activities across countries. However, these factors are inherently challenging to quantify using statistical methods. Other factors that have been identified as influencing sport participation are wealth and education [see i.e., (20)]. These factors are not government inputs designed to increase sport activity; rather, they are ends in themselves. However, we will, together with the quality of government and region/welfare system, include them in the regression model as contextual factors.

### Analyses

We have conducted a series of statistical analyses to address the research questions posed in this paper. First, we present descriptive statistics organized by region for key input variables and the outcome variables. This step provides an overview of how each country and region performs in terms of public expenditure on sport and exercise as well as participation in these activities. It also helps to establish the relevance of these variables for analyzing public policies. Second, we performed several correlation analyses to examine the strength of the relationships between these variables (R square) and to evaluate the effectiveness of public investments across different countries and regions.

Third, we conducted multivariable analysis to gain deeper insights into the factors influencing participation in sport and exercise. At this stage, we moved beyond treating each country as an isolated unit and instead focused on cross-country factors. These included contextual variables as well as sport-related input and output variables within the model, allowing for a comprehensive examination of overall performance. A detailed explanation of the variables incorporated into the model, which serves as the foundation for the linear regression analyses, is provided later in the paper. To assess the contribution and strength of each variable, we employed significance levels *t*-tests/chi-square tests, while the R-squared value was used to evaluate the overall explanatory power of the different models. Additionally, a correlation matrix was included to examine the internal relationships between the independent variables. Collinearity diagnostics were also performed to ensure the robustness and reliability of the analysis. It is important to emphasize, however, that the multivariable analyses are based on cross-sectional datasets. As such, these analyses necessitate careful interpretation, and we caution against treating the identified correlations or observed effects as definitive proof of causal relationships.

## Results

### Descriptive statistics structured by region

**Figure 1:**
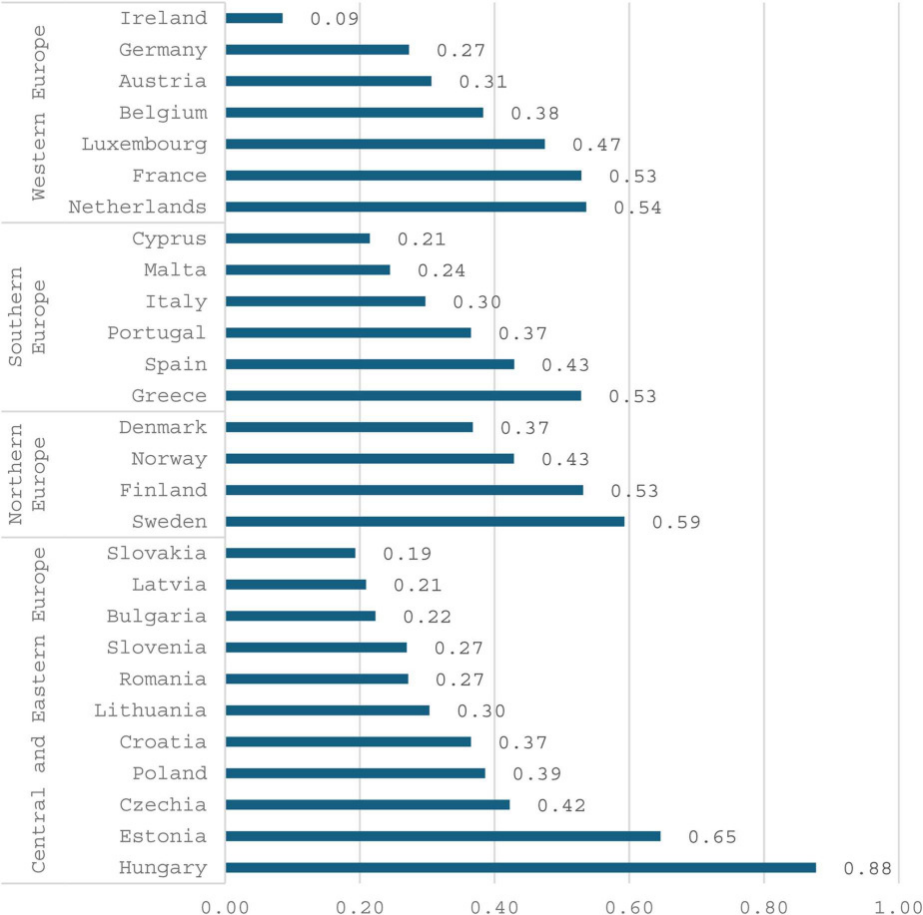
General government expenditure on recreational and sporting services in percent of GDP, 2021. Source Eurostat.

**Figure 2:**
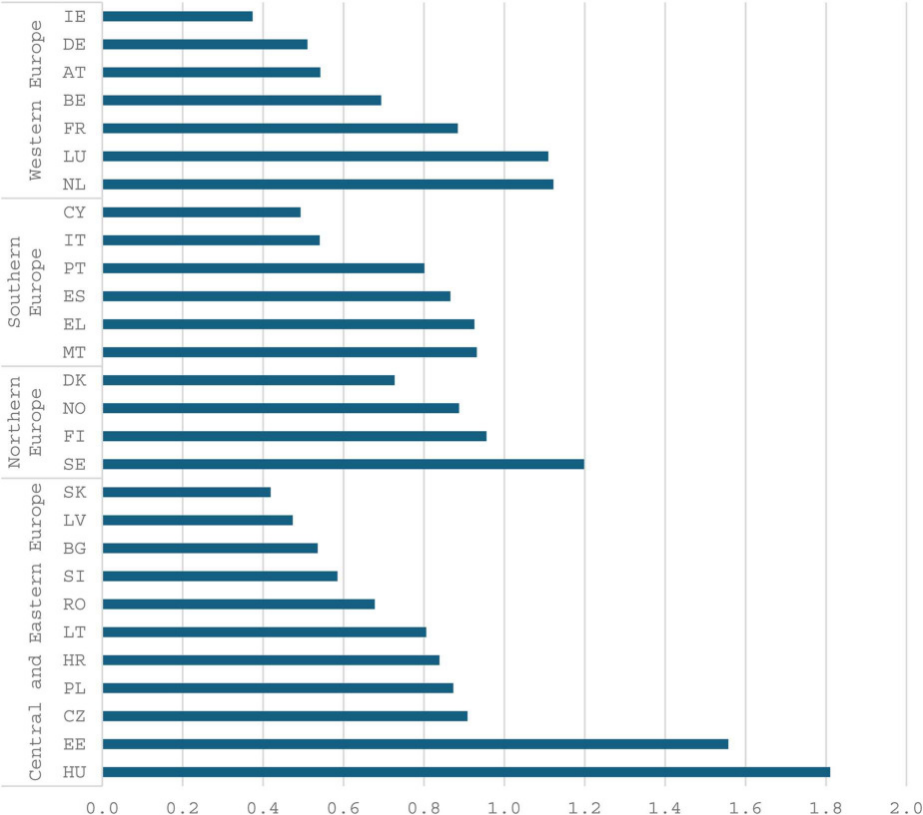
General government expenditure on recreational and sporting services in percent of total government expenditure, 2021. Source Eurostat.

**Figure 3:**
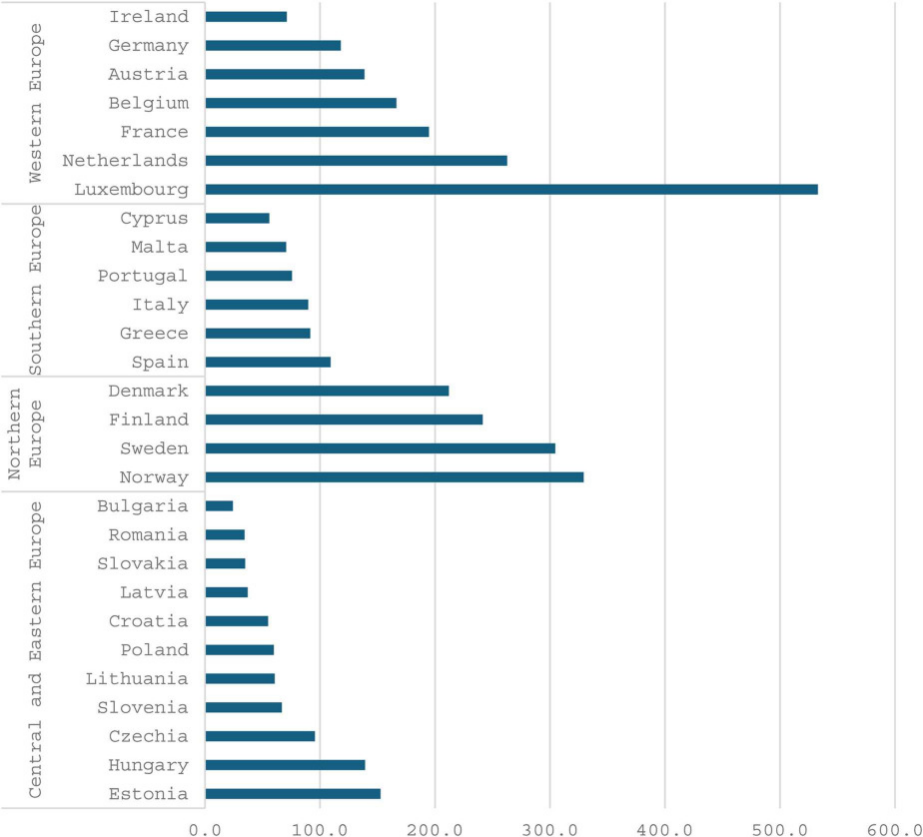
General government expenditure on recreational and sporting services in euro per inhabitants, 2021. Source Eurostat.

The figures below (Figures 1, 2 and 3) present several noteworthy findings. The findings indicate that, in the context of the overall economy, Central and Eastern European countries, such as Hungary and Estonia, prioritize sports to a greater extent than Northern and Western European countries. This observation stands in contrast to the expectation that nations in the latter group might be more inclined to support a secondary objective of the welfare state, such as sports (6). In contrast, poorer countries are expected to prioritize primary objectives, including health, education, and security. However, the data reveal a notable deviation from this expectation. Specifically, the analysis uncovers a pronounced prioritization of sports and related services within the public budget. Several relatively poorer countries in Eastern and Southern Europe allocate a higher percentage of their public budgets to sports than do more affluent countries in Western and Northern Europe. Notably, liberal economies such as Ireland are positioned towards the lower end of this spectrum.

Nevertheless, when examining public expenditure on sports and recreational services in euros per capita, it is evident that the wealthiest countries in Northern and Western Europe allocate the most substantial budgets, with the notable exception of Ireland. It is still noteworthy that relatively poorer countries, such as Hungary and Estonia, allocate more euros per capita than wealthy countries like Germany and Austria.^4^

**Figure 4:**
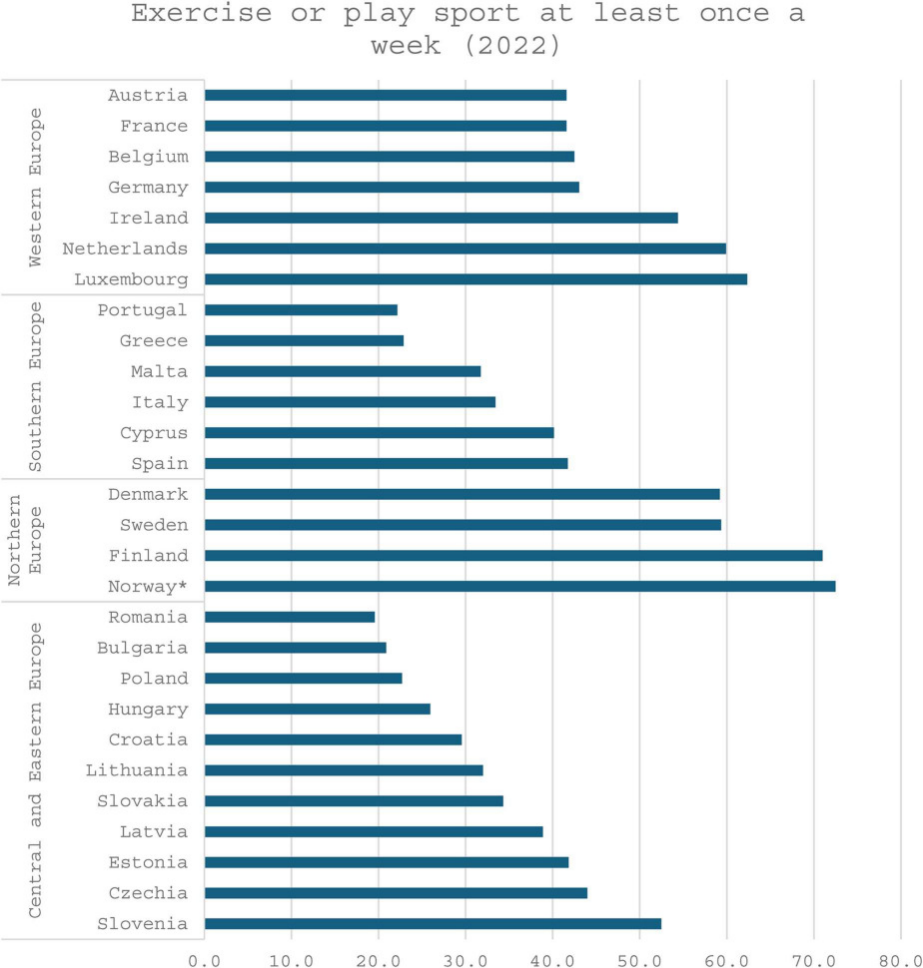
Exercise or play sport at least once a week, 2022. Percentage 15+. Source: Eurobarometer.

In the realm of sport and exercise participation, a diverse range of statistical metrics is employed, encompassing general participation in sport and exercise, attendance at sporting events, and more targeted indicators such as engagement in health-enhancing physical activities. Despite the inherent diversity of these metrics, a clear and consistent pattern emerges, as outlined in the chapter on Sport within the benchmarking sub-study of 2024 by EIPA (21). Nordic countries consistently achieve the highest rankings, followed by Western European nations, with Central, Eastern, and Southern European countries typically occupying the lower tiers. Nevertheless, notable exceptions disrupt this trend, including Slovenia, the Czech Republic, and Spain. While Hungary performs poorly in terms of overall sport and exercise participation, it demonstrates a relatively strong level of engagement in health-promoting physical activities. This apparent discrepancy can be attributed to variations in measurement approaches and conceptual interpretations. For example, the conceptualization of “sport” may as argued differ significantly between Western and Eastern European contexts (19). The figure below (Figure 4) illustrates weekly sport and exercise participation rates in 2022, serving as a representative variable that integrates these diverse metrics.

### Effectiveness of public investment—correlation analysis

**Figure 5:**
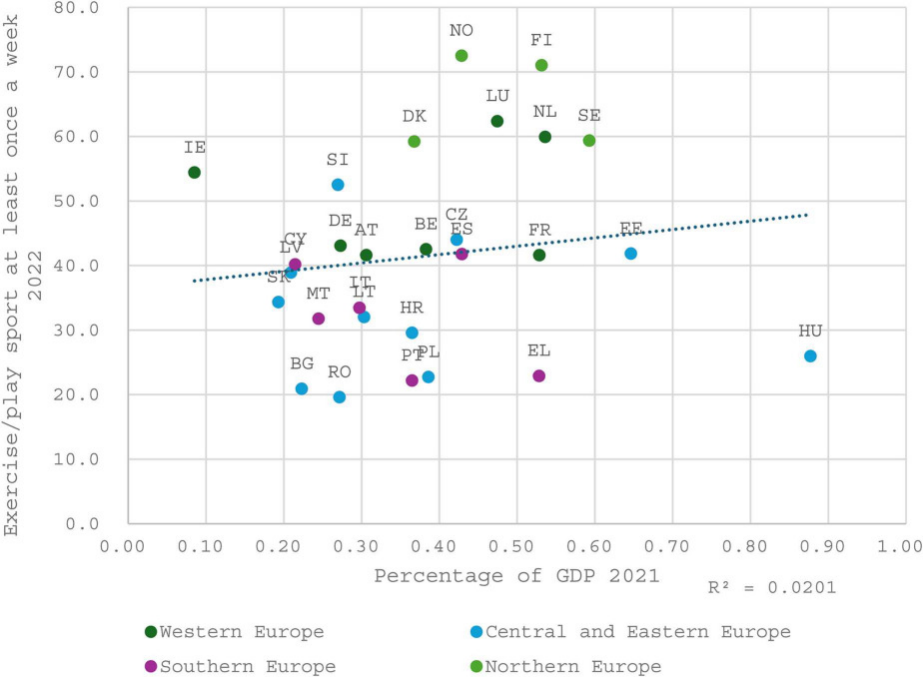
The correlation between public expenditure of percentage of GDP in 2021 and doing exercise or/and playing sport at least once a week in 2022. (Also published in (21): chp. 4, figure 25).

**Figure 6:**
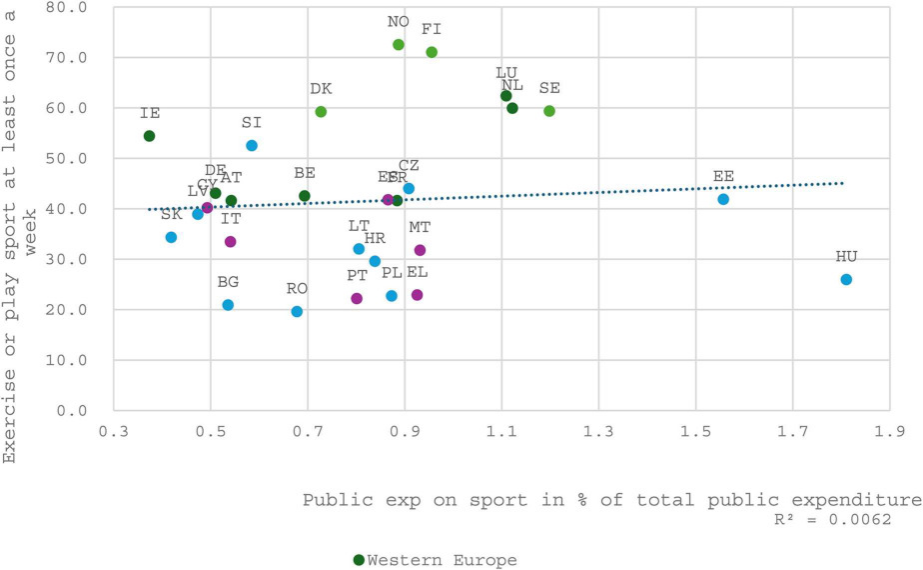
The correlation between public expenditure of percentage of total public expenditure in 2021 and doing exercise or/and playing sport at least once a week in 2022.

**Figure 7:**
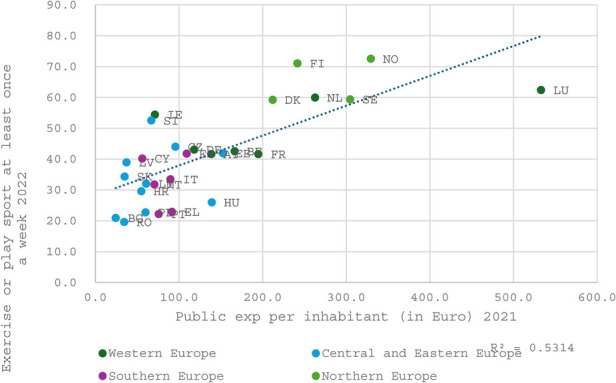
The correlation between public expenditure per inhabitant (in euro) in 2021 and doing exercise or/and playing sport at least once a week in 2022.

The following three figures (Figures 5, 6 and 7) collectively illustrate the relationship between public investment in sports and recreation and the frequency of sport or exercise participation at least once a week. The first figure highlights public priorities regarding sports and exercise relative to other sectors within the national economy. The second figure visualizes the allocation of funding for sports within national public budgets. The third figure analyzes public expenditure on sports and recreation expressed in euros per inhabitant, thereby also reflecting the influence of a nation's overall wealth on its level of investment in and support for the sports sector.

The correlation between government expenditure on sports and physical activity, expressed as a percentage of GDP, and participation rates is notably weak (R^2^ = 0.02). A straightforward interpretation of this R-squared value suggests that an increase in public expenditure on sports and exercise by 0.1 percentage points of GDP is estimated to yield only a 0.2 percentage point rise in sport and exercise participation within the population. This finding highlights the limited direct influence of government spending on sport and physical activity levels, a conclusion further reinforced by a study on the efficacy of public expenditure on sports in EU countries conducted by Nessel and Kościółek (22). In their analysis, government expenditure on sports and recreation as a percentage of GDP is utilized as an input factor (with an alternative model also incorporating private household expenditure). However, the study incorporates two output factors: mass sport participation and the number of Olympic medals won. The findings reveal that only Sweden, Slovenia, Slovakia, Malta, Lithuania, Ireland, and Finland perform efficiently, suggesting that the remaining 20 countries included in the study operate inefficiently. This inefficiency implies that these countries could potentially reduce their sport-related expenditures while maintaining—or even improving—their sporting outcomes [(22), p. 842]. Notably, there is a significant overlap between the countries identified as high-performing in this study and those that score well in our more straightforward correlation analysis, further validating the findings.

The primary role of government in the realm of sports is to enable and support activities that benefit the entire population—areas that are generally underfunded by private entities. It is essential to recognize, however, that these publicly funded initiatives often serve as foundational prerequisites for broader participation in sports and physical activity. On the other hand, it is evident that the financial burden of engaging in sports and exercise—such as membership fees, training center costs, equipment purchases, and travel expenses—rests predominantly with private households. As a result, the correlation between private spending and participation in sports and physical activity is significantly stronger [see (21), Chapter 4, Figure 27] than the correlation observed with public funding, which is the primary focus of this paper.

Despite this, one might reasonably expect a relationship between the government's prioritization of sport and recreation within the state budget and national participation rates. Yet, the data reveals an unexpectedly weak correlation, with an R-squared value of just 0.006 (see Figure 6 below). This finding highlights the minimal direct impact of public funding prioritization on overall participation rates.

This raises a critical question about the adequacy of the social return on public investment. Specifically, it remains unclear whether governments are effectively channeling resources into the construction or support of facilities and programs that genuinely promote widespread public engagement in sports and exercise. Conversely, when examining the amount of public funding allocated per capita, a much stronger relationship with participation rates emerges, reflected by an R-squared value of 0.53 (see Figure 7 below). The relationship between public funding for sports and exercise and the resulting participation rates across countries, while significant, is not entirely surprising. It could be argued that the true explanatory factor lies not in the effectiveness of sports policies themselves but rather in the overall wealth of the country, which inherently influences both funding capacity and participation levels.

Notably, the Nordic countries and the Netherlands exhibit high participation levels, consistently surpassing the correlation line in all three figures. This indicates that they may achieve a superior social return on investment compared to the European average. Whether this phenomenon is attributable to policy effectiveness or driven by contextual factors such as general wealth, cultural practices, and civic traditions remains an open question, which will be explored in the subsequent section.

### Multivariate analysis—contextual factors possible influence

As previously emphasized, public policy or investment in sports is merely one among several determinants influencing a country's participation rate. In the correlation matrix below, we have incorporated a variety of factors that could potentially impact participation rates. Naturally, there are other influential elements, for instance individual factors such as age and sex, that significantly affect activity levels (3). These factors, however, have been excluded from our analysis as they are both difficult to influence and offer limited utility for generating actionable political insights. Moreover, they tend to exhibit a high degree of uniformity across countries, thereby reducing their variability as a meaningful basis for comparison. This is not to suggest that gender differences in participation rates are unimportant—on the contrary, women tend to have higher participation rates in more gender-balanced systems (23). Additionally, while participation rates generally decline with age across all countries, Denmark, Iceland, Sweden, and Austria display a less pronounced decline (20). Nonetheless, if we do not observe significant differences in the proportion of women across countries or a markedly larger elderly population in some nations, these trends are better understood as outcomes of political regimes rather than intrinsic components of them. Furthermore, a gender-balanced system is largely encapsulated by the variables *quality of governance* and *region/political regimes* (see below).

Additionally, the level of urbanization is a pertinent factor (24). To capture its influence, we have chosen to include access to recreational and green areas as an intermediate output factor, serving as a proxy for the degree of urbanization. Further, the inclusion of regions in Europe may reflect various climate condition that can influence the participation level. The core of the model is however composed of well-established contextual variables, including wealth, educational attainment, and quality of governance (QoG).^5^ These factors are assumed to significantly influence physical activity levels and can be effectively evaluated at the national level. Additionally, we have incorporated sports-related factors, both input and output, as outlined in the chapter on sports in the EIPA (21) benchmarking report.

These factors can be categorized into three distinct groups:

1. Background or contextual variables: These are structural elements related to the country that aren't specific to sports, such as economic conditions, educational level or geographical location.
2. Intermediate input factors: These represent the resources allocated to sports, including funding and infrastructure investments.
3. Intermediate output factors: These are variables within the sports sector that result from the input factors, such as the availability of recreational facilities and membership levels in sports clubs.

This structured approach allows for a comprehensive understanding of the various influences on participation rates (see Appendix Table A1). Firstly, it is evident that there is a relatively strong internal correlation among the background factors included in the analysis. Exceptions to this pattern are found in the general GDP and the percentage of GDP allocated to sport and recreation, both of which exhibit weak or insignificant correlations with contextual variables and intermediate factors related to the sport and exercise sector. Furthermore, it is noteworthy that while public investment in sport, expressed as a percentage of GDP, correlates with public expenditure in sport and exercise per inhabitant, it shows weak or no correlation with employment in the sports sector. In addition, expenditure per capita positively correlates with output variables such as access to recreational areas and membership in sports clubs. Concerning our independent (outcome) variable, we observe that all factors, except for general GDP and the percentage of GDP spent on sport and recreation, positively correlate with participation rates. Notably, the quality of government displays a particularly strong correlation.

The regression analysis (Table 1) further revealed that the quality of governance has a significant correlation with participation rates across various countries, even when accounting for other contextual and sport-related variables within the models.^6^ Perhaps the most striking finding is that the models account for a considerable portion of the variance in participation rates among the countries studied. Moreover, incorporating additional sport-related input and output factors does not enhance the explanatory power of the models, suggesting that contextual factors are predominantly influential in determining participation rates. This observation aligns with the findings of Nessel and Kościółek (22).

**Table 1:** Linear regression analyses. Dependent variable: Exercise or play sport once a week in 2022.

	Model1	Model2	Model3
Variables	Beta-coefficient	Beta-coefficient	Beta-coefficient
Real GDP per capita (Euro), 2022	0,39*	0,30	−0,16
% holding a tertiary education (levels 5–8), 2022	0,09	0,10	−0,12
European Quality Index (1–100), 2017	0,55^**^	0,53**	0,47*
Dummy: 0 = Northern Europe; 1 = Western Europe	−0,36^**^	−0,35*	−0,11
Dummy: 0 = Northern Europe; 1 = Central and Eastern Europe	−0,11	−0,11	0,10
Dummy: 0 = Northern Europe; 1 = Southern Europe	−0,27	−0,25	−0,04
% of GDP		−0,05	−0,24
Public exp per inhabitant (in Euro) 2021		0,13	0,56
Percentage of total employment in 2022			0,32
Access to recreational or green areas (2016). Rather or very difficult			−0,23
Member of a sports club, fitness/health club or social organization for sport (2022)			0,00
(Constant)	14,7	16,2	33,0
Adjusted R^2^ (model sig.)	0,749(<0,001)	0,726(<0,001)	0,720(<0,001)

* sig = 0,1; ^**^sig = 0,05

When sports-related factors are integrated into the model, the influence of a country's wealth and education levels diminishes. This highlights the intricate interplay among several independent variables, as illustrated in Appendix Table A1, where overlapping effects are distributed across multiple variables. As a result, the significance of each individual variable is attenuated. Moreover, this suggests that sport-sector-related variables primarily act as intermediary factors, shaped by broader contextual elements that determine the structure of the sports sector, which, in turn, influences participation levels.

Nonetheless, sport-sector-related variables also exhibit an independent association with participation rates. For instance, factors such as public spending on sports (measured in euros), the percentage of the workforce employed in the sports sector, and access to recreational spaces show positive correlations with participation levels, even after accounting for contextual variables. However, these correlations tend to be relatively weak.

## Discussion

Seeing this as a study of the EU countries as such, the findings suggest that investments in the sports sector—perhaps unsurprisingly—have some impact on participation rates. However, the prioritization of the sports sector as a percentage of GDP demonstrates no significant correlation with participation rates. This indicates that while targeted investments in the sports sector may influence participation, merely allocating a larger share of GDP to the sector does not necessarily translate into higher engagement in sports activities.

Once again, the most striking finding is the pivotal role of governance quality. Countries characterized by high levels of trust in public institutions and effective governance consistently exhibit higher participation rates, regardless of their spending on sports. This underscores that it is not merely the amount of financial investment that matters, but the quality of policy design and implementation.

This finding underscores the importance of expanding the analytical framework to include contextual factors that extend beyond the confines of the current model, such as cultural dynamics and deeply embedded institutional characteristics. This perspective aligns with the insights of Nicholson et al. (10) and Willem and Scheerders (11). Moreover, other policy domains—such as transport policies and urban planning—play a critical, albeit often understated, role in shaping physical activity patterns. Norway offers a compelling example, despite not being included in this study. While Norwegians display high participation rates in sport and exercise, they rank lower in active transportation behaviors, such as walking or cycling to school or work, and fall short in achieving adequate overall physical activity levels (10).^7^ This highlights the limitations of wealth or educational attainment in fully compensating for deficiencies in infrastructure and transportation systems, further suggesting that culture may serve as a pivotal, yet often overlooked, factor. Culture, often neglected in discussions on physical activity, both influences and is influenced by policy. It shapes behaviors by fostering environments that encourage physical activity while simultaneously accommodating paradoxical practices, such as driving to the gym.

Can we identify a shared cultural characteristic that accounts for the high participation rates in sport and exercise observed in the Nordic countries and Western European nations such as Germany, the Netherlands, and Ireland (6, 21, 26, 27)? While these countries differ in political regimes—drawing on Esping-Andersen's classification of social democratic, conservative, and liberal welfare states [see (6, 21)]—as well as in religious traditions (Protestant and Catholic) and geographical contexts (*N*orthern Europe, Continental Europe, and Western Europe), one prominent feature stands out: a robust civic tradition and culture, often rooted in the 19th century or earlier, appears to be the most significant factor in explaining their success.

This is exemplified by Finland's (28, 29, 36) long-standing tradition of civic engagement, where sports were often intertwined with broader social movements; Ireland's (30) strong local anchoring and community involvement, juxtaposed with a historically laissez-faire approach from central authorities regarding sports; and the Netherlands' (16, 31) focus on a vibrant voluntary movement supported by independent and resilient clubs. In stark contrast, former Eastern Bloc countries such as Lithuania (32), Macedonia (33), and Hungary (38) exhibit weaker civic traditions alongside a pronounced state-bureaucratic legacy. Similarly, Southern European nations emphasize the family as the cornerstone of society (21), a cultural orientation that differs significantly from the civic structures found in the Nordic and Western European countries. However, fostering a strong civic culture and tradition is challenging to achieve politically, particularly in the short term.

This cultural legacy is further institutionalized within the structural organization of the sport sector, as outlined in the background section. In Nordic and Western European countries, the voluntary sector plays a more prominent role, characterized by a strong club network supported by substantial public funding, in contrast to the structures observed in Central, Eastern, and Southern European nations (12, 13). Nonetheless, caution is warranted when interpreting this directly, as the majority of sport and exercise activities occur outside formal organizational frameworks.

Although public investment in sport and exercise exhibits a weak correlation with participation rates, it would be premature to conclude that public policies have no impact. There may be indirect effects stemming from the content and composition of these policies that are not fully captured in the current analysis. In-depth qualitative studies could play a crucial role in uncovering these nuanced dynamics. Moreover, policies aimed at increasing physical activity across various sectors—such as education, transportation, and urban infrastructure—may exert a significant influence on activity levels (34). Additionally, research by Volf et al. (35, 37) highlights the potential of targeted policy actions, such as constructing sports facilities and reducing financial barriers, to boost sports participation. However, a persistent challenge remains: broad, overarching programs often fail to effectively engage the least active segments of the population. Addressing this issue is critical to ensuring that policy initiatives are inclusive and capable of reaching those who would benefit most from increased physical activity.

## Conclusion

This study acknowledges several limitations. As highlighted in the methos section, caution is advised when interpreting the figures directly, especially concerning self-reported physical activity data. The reliability of surveys varies across countries, and the understanding of 'sport and exercise’ differs, alongside a propensity for individuals to overestimate their activity levels—which may not uniformly vary between nations. Further, since this is based on cross-sectional datasets one should be reluctant to draw causal conclusions.

Nonetheless, several key findings warrant attention and should serve as foundational stepping stones for further in-depth analysis. As previously emphasized, the quality of governance emerges as the most significant variable in terms of explanatory power. Notably, this variable aligns closely with the type of welfare regime, as the degree of trust citizens place in the public sector appears to be a defining characteristic of Social Democratic and partly Conservative welfare systems. While the direct impact of public policy on sport and exercise participation may be limited, the broader actions of governments remain highly influential, even when controlling for variables such as wealth, education, and geographic region. This underscores the critical role of effective governance in fostering environments conducive to increased physical activity.

Additionally, to fully understand the factors shaping a population's activity patterns, it is essential to look beyond contemporary variables. Historical, cultural, and institutional legacies established long before the emergence of current welfare regimes may hold valuable insights into the underlying drivers of participation. These deeper structural and cultural influences merit closer examination to uncover the enduring factors that influence engagement in sport and exercise.

## Data Availability

Publicly available datasets were analyzed in this study. This data can be found here: https://ec.europa.eu/eurostat; https://europa.eu/eurobarometer; https://www.eurofound.europa.eu.

## Footnotes

^1^It is important to note that this analysis is based on data from only ten European countries, which underscores the need for caution when drawing definitive conclusions. The countries included in the study are Spain, Norway, Poland, England, Denmark, Switzerland, Belgium (Flanders), Spain, Germany and the Netherlands.

^2^ https://ec.europa.eu/eurostat/statistics-explained/index.php?title=Government_expenditure_on_recreational_and_sporting_services

^3^Eurostat is by and large based on extraction for public sources/register in each country, while Eurobarometer and Eurofund are based on surveys conducted in each country.

^4^General government expenditure refers to the total expenditures from all levels of the public sector in a country, including the central government, regional authorities, and local governments. However, the question remains as to whether the entirety of expenditures in for instance the Länder of Germany, which are responsible for a significant portion of the sport and exercise sector, are adequately captured in the available statistics.

^5^The QoG-index is based on a large citizen survey where respondents are asked about perceptions and experiences with public sector corruption, along with the extent to which citizens believe various public sector services are impartially allocated and of good quality (see 25).

^6^Although we have included the standardized coefficients (beta) in the table, caution is advised against interpreting them directly.

^7^EIPA (Forthcoming). Update chapter on Sport, in *Public Sector Performance Programme 2022-2025. Benchmark Study update 2025*. Maastricht: European Institute of Public Administration.

## Appendix

**Table A1:** Correlation matrix of the included variables.

	European Quality Index (1–100), 2017	Population by educational attainment level, tertiary education (levels 5–8), 2022	GDP (2022-Q4), current prices, million euro	Real GDP per capita (Euro), 2022	% of GDP	Public exp per inhabitant (in Euro) 2021	Percentage of total employment in 2022	Access to recreational or green areas (2016). Rather or very difficult	Member of a sports club, fitness/health club or social organisation for sport (2022)
Context variables									
European Quality Index (1–100), 2017									
Population by educational attainment level, tertiary education (levels 5–8), 2022	,670^**^								
GDP (2022-Q4), current prices, million euro	0,218	−0,115							
Real GDP per capita (Euro), 2022	,751**	,640**	0,150						
Sport related input variables									
% of GDP	0,133	0,042	0,003	−0,029					
Public exp per inhabitant (in Euro) 2021	,681**	,507**	0,072	,730**	,497**				
Sport related output variables									
Percentage of total employment in 2022	,543**	,590**	0,050	,642**	−0,003	0,240			
Access to recreational or green areas (2016). Rather or very difficult	-,721**	-,726**	−0,091	-,572**	−0,095	-,516**	-,477*		
Member of a sports club, fitness/health club or social organisation for sport (2022)	,725**	,440*	0,136	,704**	0,054	,531**	,553**	-,644**	1
Outcome—dependent variable									
Exercise or play sport at least once a week 2022	,845**	,637**	0,076	,734**	0,132	,690**	,569**	-,755**	,717**

*sig = 0,1; **sig = 0,05.
